# Use of Ballistocardiography to Monitor Cardiovascular Hemodynamics in Preeclampsia

**DOI:** 10.1089/whr.2020.0127

**Published:** 2021-04-20

**Authors:** Odayme Quesada, Md Mobashir Hasan Shandhi, Shire Beach, Sean Dowling, Damini Tandon, James Heller, Mozziyar Etemadi, Shuvo Roy, Juan M. Gonzalez Velez, Omer T. Inan, Liviu Klein

**Affiliations:** ^1^ Women's Heart Center, The Christ Hospital Heart Vascular and Lung Institute, Cincinnati, Ohio, USA.; ^2^ Barbra Streisand Women's Heart Center, Cedars-Sinai Smidt Heart Institute, Los Angeles, California, USA.; ^3^ School of Electrical and Computer Engineering, Georgia Institute of Technology, Atlanta, Georgia, USA.; ^4^ Division of Cardiology, Department of Internal Medicine, University of California San Francisco, San Francisco, California, USA.; ^5^ Department of Obstetrics and Gynecology, University of California San Francisco, San Francisco, California, USA.; ^6^ Department of Anesthesiology, Northwestern University, Chicago, Illinois, USA.

**Keywords:** ballistocardiography, cardiovascular hemodynamics, hypertensive disorders of pregnancy, preeclampsia, pregnancy, women

## Abstract

***Objective:*** Pregnancy requires a complex physiological adaptation of the maternal cardiovascular system, which is disrupted in women with pregnancies complicated by preeclampsia, putting them at higher risk of future cardiovascular events. The measurement of body movements in response to cardiac ejection *via* ballistocardiogram (BCG) can be used to assess cardiovascular hemodynamics noninvasively in women with preeclampsia.

***Methods:*** Using a previously validated, modified weighing scale for assessment of cardiovascular hemodynamics through measurement of BCG and electrocardiogram (ECG) signals, we collected serial measurements throughout pregnancy and postpartum and analyzed data in 30 women with preeclampsia and 23 normotensive controls. Using BCG and ECG signals, we extracted measures of cardiac output, J-wave amplitude × heart rate (J-amp × HR). Mixed-effect models with repeated measures were used to compare J-amp × HRs between groups at different time points in pregnancy and postpartum.

***Results:*** In normotensive controls, the J-amp × HR was significantly lower early postpartum (E-PP) compared with the second trimester (T2; *p* = 0.016) and third trimester (T3; *p* = 0.001). Women with preeclampsia had a significantly lower J-amp × HR compared with normotensive controls during the first trimester (T1; *p* = 0.026). In the preeclampsia group, there was a trend toward an increase in J-amp × HR from T1 to T2 and then a drop in J-amp × HR at T3 and further drop at E-PP.

***Conclusions:*** We observe cardiac hemodynamic changes consistent with those reported using well-validated tools. In pregnancies complicated by preeclampsia, the maximal force of contraction is lower, suggesting lower cardiac output and a trend in hemodynamics consistent with the hyperdynamic disease model of preeclampsia.

## Key Messages

- **What is already known about this subject?** Pregnancy requires a complex physiological adaptation of the maternal cardiovascular system, which is disrupted in women with pregnancies complicated by preeclampsia.
- **What does this study add?** In pregnancies complicated by preeclampsia, the maximal force of contraction is lower, suggesting lower cardiac output and **a** trend in hemodynamics consistent with the hyperdynamic disease model of preeclampsia.
- **How might this impact on clinical practice?** Using ballistocardiography, we noninvasively measured cardiac hemodynamics throughout pregnancy in the clinical setting similar to the time it takes to obtain vital signs.

## Introduction

Preeclampsia, which complicates up to 10% of pregnancies worldwide, results in a fourfold higher risk of heart failure (HF) and a sixfold higher risk of cardiovascular death in comparison with women with normotensive pregnancies.^1–3^ Preeclampsia is characterized by the development of *de novo* hypertension after 20 weeks of gestation along with systemic manifestations.^4^ Pregnancy requires a complex physiological and hemodynamic adaptation of the maternal cardiovascular system, which is disrupted in women with pregnancies complicated by preeclampsia.

Cardiac ejection of blood into the aorta generates small repetitive displacements of the center of the mass of the body with each heartbeat yielding a ballistocardiogram (BCG) signal that can be measured externally.^5^ A more recent work based on mechanistic modeling has elucidated that the BCG may originate from blood pressure gradients in the ascending and descending aorta.^6^ The movement of the center of mass of the body (*i.e.*, BCG) can be recorded externally with a modified weighing scale,^7^ bed-mounted sensors,^8^ and modified toilet seat.^9^ In our previous works with a weighing scale-based BCG recording system, we modified an electronic weighing scale to measure the BCG signal,^7^ which consists of waves that correspond to aortic valve opening and closing and rapid ejection of blood into the aorta on echocardiography.^5^

The J-wave amplitude (J-amp) on the BCG has been correlated with relative changes in stroke volume and thus, when multiplied by the heart rate (HR), can be used as a surrogate for relative changes in cardiac output. In healthy volunteers recovering from treadmill exercise, the percentage change in the BCG signal was strongly correlated with the percentage change in cardiac output measured by Doppler echocardiogram.^10^ In our previous works in patients with HF, we have also shown the effectiveness of the modified weighing scale-based BCG in tracking hemodynamics and clinical status of patients with HF.^11,12^ Given the implications of preeclampsia on cardiovascular disease, particularly HF, the ability to noninvasively monitor cardiovascular hemodynamic changes during pregnancy and postpartum is of great value.^13^

This pilot study was a multidisciplinary collaborative effort to prospectively examine the effectiveness of a modified weighing scale-based BCG monitoring system in tracking changes in intracardiac hemodynamics throughout pregnancy and postpartum in pregnancies complicated by preeclampsia in comparison with normotensive pregnancies as controls. This work provides the foundation for future studies using a noninvasive device to detect cardiac hemodynamic changes through signals when increased clinical surveillance is needed during and after a pregnancy complicated by preeclampsia.

## Methods

### Trial design and oversight

This observational, prospective, longitudinal cohort study was conducted under a protocol reviewed and approved by the University of California, San Francisco (UCSF), and Georgia Institute of Technology Institutional Review Board (IRB). All women enrolled provided written consent. Because this was a pilot study, a trial management group and independent trial steering and data monitoring committees were not established. Patients suitable for enrollment were recruited and entered into an observation, prospective pilot study, for a total of 92 pregnant women, at the University of California, San Francisco Mission Bay Hospital. Data supporting our findings are available upon reasonable request from the corresponding author.

### Patient involvement

Patients were involved in the conduct of this research. During the feasibility stage, the priority of the research question, choice of outcome measures, and methods of recruitment were informed by discussions with patients. Once the trial has been published, participants will be informed of the results and will be sent details of the results in a study newsletter suitable for a nonspecialist audience.

### Study visit

Pregnant women were enrolled in the obstetrics clinic or labor and delivery unit. Women with normotensive pregnancies were recruited during the first trimester (T1) of pregnancy as well as a subset of women at high risk of developing preeclampsia, including a history of preeclampsia in prior pregnancies, elevated blood pressure, or hypertension in T1. A separate cohort of women with clinical suspicion or diagnosis of preeclampsia were recruited during the second trimester (T2) or third trimester (T3) of pregnancy. After enrollment, all women were followed longitudinally, and BCG and electrocardiogram (ECG) measurements were obtained throughout pregnancy during obstetrician clinic visits, hospitalizations before and after delivery, and postpartum clinic visits. For a subset of women with normotensive control pregnancies and preeclampsia, an additional postpartum study visit >180 days after delivery was completed. There was no limit on the number of measurements obtained per subject, and efforts were made to obtain measures after enrollment at each prenatal visit and/or daily when hospitalized. T1 was defined as 0–13 weeks' gestation, T2 as 14–26 weeks' gestation, and T3 as 27–40 weeks' gestation. Postpartum measurements were divided into three groups: immediate postpartum (I-PP) was defined as 0–6 days postpartum, early postpartum (E-PP) as 7–180 days postpartum, and late postpartum (L-PP) as >180 days postpartum.

For all women, BCG and simultaneous ECG measurements were obtained by having the women stand on the modified bathroom scale as still as possible for 1–2 minutes for a total of 4 recordings per visit, including two recordings while taking deep breaths. For hospitalized patients, measurements were made when patients were hemodynamically stable and able to stand safely on the scale. Blood pressure was taken at the time of BCG/ECG measurements and averaged for each individual patient. Blood pressure medications and doses given on the day of BCG/ECG measurements were recorded.

At study completion, a chart review was completed to determine preeclampsia diagnosis based on the 2013 ACOG Task Force on Hypertension in Pregnancy guidelines.^4^ Preeclampsia was defined as *de novo* hypertension (≥140/90) during pregnancy after 20 weeks of gestation and proteinuria (≥300 mg protein in 24-hour urine collection or ≥0.3 protein/creatinine ratio) or, in the absence of proteinuria, presence of thrombocytopenia (platelet count less than 100,000/μL); impaired liver function (elevated blood levels of liver transaminases to twice the normal concentration); new development of renal insufficiency (elevated serum creatinine greater than 1.1 mg/dL or doubling of serum creatinine in the absence of other renal diseases); pulmonary edema; or new-onset cerebral or visual disturbances. Severe preeclampsia was defined as systolic blood pressure ≥160 mmHg or diastolic blood pressure ≥110 mmHg; hypertension requiring antihypertensive therapy; thrombocytopenia (platelet count less than 100,000/μL); impaired liver function (elevated blood levels of liver transaminases to twice the normal concentration); new development of renal insufficiency (elevated serum creatinine greater than 1.1 mg/dL or doubling of serum creatinine in the absence of other renal diseases); pulmonary edema; or new-onset cerebral or visual disturbances. Superimposed preeclampsia was defined as chronic hypertension that predated pregnancy and development of preeclampsia during pregnancy. Preeclampsia diagnosis was verified by a maternal–fetal medicine expert (Juan M. Gonzalez Velez, MD, PhD). Baseline characteristics and demographics were also obtained from the medical record based on questionnaires completed in the obstetrics clinic or hospitalization.

### Hardware design

**FIG. 1.**
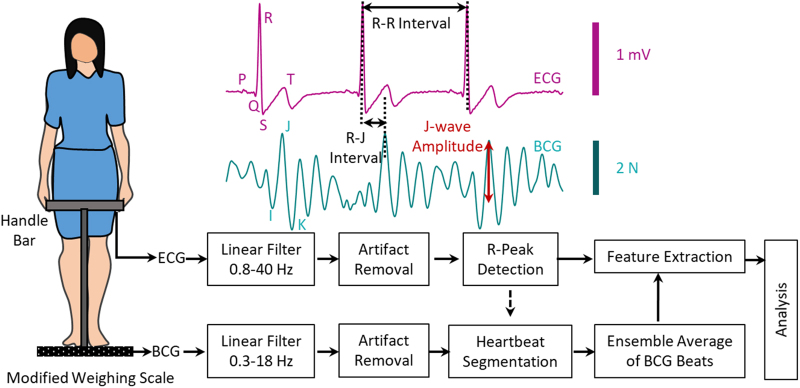
Diagram of the experimental setup and processing steps.

The BCG signal was measured with a modified bathroom scale, and the ECG signal was measured with handlebar electrodes interfaced with custom electronics.^7,14^ Figure 1 shows a diagram of the experimental setup and processing steps. The signals were sampled at 1 kHz and recorded on a secure digital card in the scale and later accessed *via* a laptop for further signal processing in MATLAB (2017a).

### Data processing and feature extraction

The raw BCG and ECG signals were filtered with finite impulse response Kaiser window band-pass filters. Cutoff frequencies for the BCG and ECG were 0.3–18 and 0.8–40 Hz, respectively. After filtering, both the signals were truncated in time to remove movement artifacts, and the R-wave peaks were located on the ECG signal. Both ECG and BCG signals were then segmented according to these R-wave peaks, and ensemble-averaged ECG and BCG heartbeats were computed from these segmented beats.^7,14^ The following features were then extracted from the averaged waveforms: J-amplitude (J-amp) and J-amp × HR. Visit measures were excluded if data quality was poor. J-amp, as a surrogate for stroke volume, and J-amp × HR, as a surrogate for cardiac output, were used for further analysis.

### Statistical analyses

**FIG. 2.**
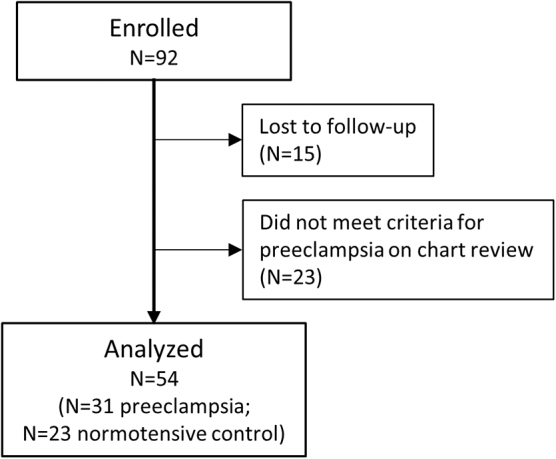
Flow chart of women enrolled and included in the analysis.

A total of 38 women were excluded from the final analysis due to loss to follow-up, defined as women with only prepartum measurements (*N* = 15), or criteria for the diagnosis of preeclampsia or gestational hypertension not being met based on ACOG guidelines on chart review at study completion (*N* = 23) (Fig. 2).

The demographics of participants between groups of normotensive controls and preeclampsia were compared using an unpaired *t*-test, assuming unequal variances. We compared J-amp × HRs between groups of normotensive controls and preeclampsia at different time points during pregnancy (T1, T2, and T3) and postpartum (I-PP, E-PP, and L-PP). For women with multiple recordings at any of the time points, all measures were averaged to have one measurement per subject per time point.

We used mixed-effect models with repeated measures to compare J-amp × HRs between groups at different time points in pregnancy and postpartum. The models included a random effect for each subject using an unstructured covariance matrix, with recording time as the within-subject factor and group as the between-subject factor. Different models were compared for random effects, including random intercept, random slope, and both random intercept and random slope in the initial analysis. Finally, the random intercept for each subject was included in the final model based on the initial analysis. *Post hoc* tests were performed with Bonferroni correction to determine the difference in J-amp × HRs between the groups at each time point during pregnancy and postpartum and differences in J-amp × HRs between time points within the groups. Results are expressed as mean values, 95% confidence intervals, and *p*-values obtained after adjustments; *p*-values below 0.05 were considered statistically significant for all analyses. All statistical analyses were performed using IBM SPSS 24.

## Results

The preeclampsia group comprised 6 (19%) women with preeclampsia, 18 (56%) women with severe preeclampsia, and 8 (25%) women with chronic hypertension with superimposed severe preeclampsia. A total of 6 women recruited in the T1 developed preeclampsia. Baseline clinical characteristics were different between normotensive controls and preeclampsia cases (Table 1). Women with preeclampsia had significantly higher prepregnancy body mass index (BMI) and higher rates of *in vitro* fertilization (IVF). We also found more preterm delivery rates in women with preeclampsia and lower gestational age and infant weight at delivery. Systolic and diastolic blood pressure levels were significantly higher in the preeclampsia women (Supplementary Table S1). Antihypertensive medications, including labetalol, nifedipine, hydralazine, and metoprolol, were given to 73% of women with preeclampsia and magnesium sulfate to 75%.

**Table 1.** Demographic and Clinical Characteristics in Preeclampsia and Normotensive Control Pregnancies

Demographic and clinical characteristics	Normotensive controls (N = 23)	Preeclampsia (N = 30)	p
Maternal age (years)	33.5 ± 2.4	34.0 ± 6.6	0.7
Race/ethnicity, *n* (%)			
White/non-Hispanic	10 (53)	12 (41)	0.8
Hispanic/Latin	4 (21)	8 (28)	0.4
Asian/Pacific Islander	5 (17)	4 (14)	0.4
Other	0	5 (17)	
Marital status, *n* (%)			
Married	17 (94)	21 (72)	0.8
Single	1 (6)	8 (28)	0.03
Education (years)	16.0 ± 1.6	14.6 ± 2.4	0.02
Gravida, *n* (%)			
G1	10 (43)	13 (43)	1.0
G ≥ 2	9 (39)	9 (30)	0.5
Prepregnancy BMI (kg/m^2^)	22.7 ± 3.9	27.3 ± 5.3	0.001
Gestational diabetes, *n* (%)	1 (5)	7 (24)	0.06
IVF, *n* (%)	0	5 (17)	0.04
Multiparity, *n* (%)	2 (11)	6 (21)	0.3
Medication use in the third trimester, *n* (%)			
Antihypertensives^a^	0	16 (73)	<0.001
Magnesium sulfate	1 (5)	22 (75)	<0.001
Preterm delivery	2 (11)	16 (55)	<0.001
Infant gestational age at delivery (weeks)	39 ± 2	35 ± 4	<0.001
Infant weight at delivery (g)	3410.2 ± 887.6	2437.0 ± 811.5	<0.001
Systolic blood pressure (mmHg)	111 ± 10	128 ± 12	<0.001
Diastolic blood pressure (mmHg)	66 ± 7	77 ± 10	<0.001

^a^ Antihypertensives include labetalol, nifedipine, hydralazine, and metoprolol.

BMI, body mass index; IVF, *in vitro* fertilization.

**FIG. 3.**
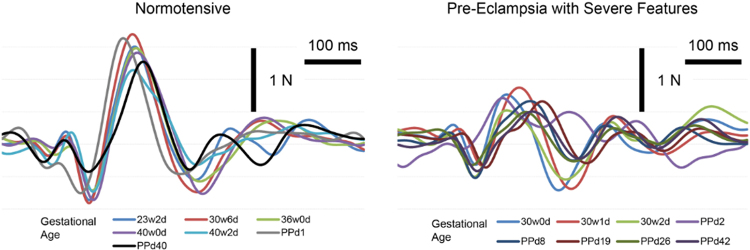
Ballistocardiogram signal during pregnancy for a woman with normotensive pregnancy and a woman with preeclampsia with severe features.

Figure 3 illustrates the serial measurement of BCG during pregnancy and postpartum for a woman with normotensive pregnancy and a woman with preeclampsia with severe features. In the pregnancy complicated by preeclampsia with severe features, the BCG shows a lower J-amp, suggesting a decreased cardiac output during pregnancy and postpartum in all the recordings at different time points during pregnancy and postpartum. The mean number of recordings for all participants was 6.5 ± 3.5, with a range of 2–18 recordings obtained for each participant. The number of recordings per trimester and postpartum time period is shown in Supplementary Table S2.

**FIG. 4.**
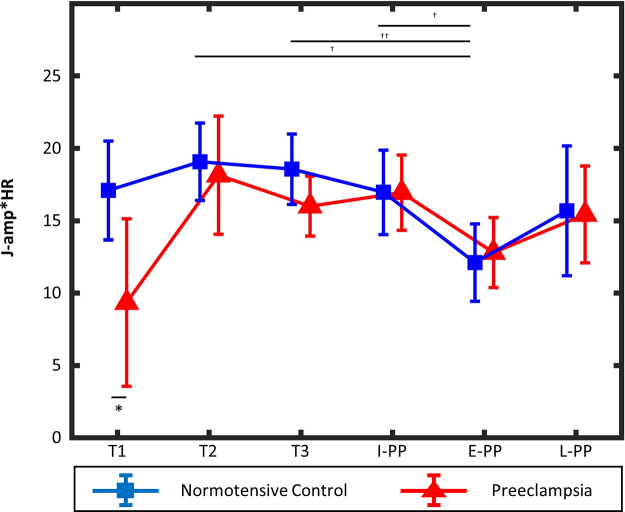
J-amp × HRs in pregnancy by trimester and postpartum for women with preeclampsia and normotensive controls. Changes in J-amp × HRs (surrogate for cardiac output) in normotensive controls and preeclampsia cases throughout pregnancy (T1, T2, and T3) and postpartum (I-PP, E-PP, and L-PP). Error bars show the standard deviation around the mean value at each time point for each group. *p < 0.05 between normotensive controls and preeclampsia groups. ^†^*p* < 0.05 and ^††^*p* < 0.01 among different recording time points in the normotensive control group. E-PP, early postpartum; HR, heart rate; I-PP, immediate postpartum; J-amp, J-wave amplitude; L-PP, late postpartum; T1, first trimester; T2, second trimester; T3, third trimester.

Using the mixed-effect model with repeated measures to compare J-amp × HRs between women with normotensive control pregnancies and preeclampsia, we found that only the recording time point main effect was statistically significant (*p* = 0.001), whereas group's main effect and the interaction effect between the two main effects were not statistically significant (*p* = 0.18 and *p* = 0.17, respectively). *Post hoc* analyses comparing normotensive controls and women with preeclampsia at different time points in pregnancy and postpartum showed significantly lower J-amp × HRs in preeclampsia cases compared with normotensive controls at T1 (*p* = 0.026) (Fig. 4). *Post hoc* analysis comparing changes at different time points in pregnancy and postpartum for normotensive controls showed that the J-amp × HR was significantly lower at E-PP compared with T2 (*p* = 0.016), T3 (*p* = 0.001), and I-PP (*p* = 0.031). No statistically significant differences in J-amp × HRs were found between time points in the preeclampsia group. In the preeclampsia group, there was a trend toward an increase in the J-amp × HR from T1 to T2 and then a drop in J-amp × HR at T3 and further drop at E-PP.

## Discussion

Using a modified weighing scale, we present (for the first time) serial cardiac hemodynamic changes during pregnancy and postpartum for women with preeclampsia and normotensive controls. In normotensive pregnancies, J-amp × HR changes in pregnancy and postpartum are consistent with validated tools. Women with preeclampsia had a significantly lower maximal force of contraction (J-amp × HR) compared with normotensive pregnancies, suggesting lower cardiac output. Although there were no significant changes in the J-amp × HR during pregnancy or the postpartum period in preeclamptic pregnancies, we observed a trend in J-amp × HRs in women with preeclampsia consistent with the hyperdynamic disease model of preeclampsia.

Using this device, in the normotensive control group, we observe changes in the J-amp × HR through the three trimesters of pregnancy and postpartum, which are consistent with the trends in cardiac output previously reported using validated tools such as echocardiography.^15–19^ To accommodate to the high volume and low resistance state of pregnancy, the maternal cardiovascular system increases cardiac output by 24 weeks' gestation in normotensive pregnancies, which is consistent with the pattern we observe in our study using ballistocardiography.^15–19^ After delivery, the cardiac output begins to decrease within the first hour to reach baseline levels by two weeks postpartum.^18^ Similarly, we find that the T2 and T3 measures of J-amp × HR were significantly higher than E-PP in normotensive controls.

Women with preeclampsia had lower education levels, higher BMI, higher use of IVF, higher use of antihypertensive medications, and infants with lower gestational age and weight, similar to prior studies.^20–22^ We observed a trend toward a lower maximal force of contraction, suggesting lower cardiac output in women with preeclampsia compared with normotensive controls during pregnancy, which was significant in the T1. These findings support the earlier studies, which found lower cardiac output in severe forms of preeclampsia, such as early-onset preeclampsia and in pregnancies complicated by small-for-gestational-age neonates, in comparison with normotensive pregnancies using validated methods.^22–24^ Similarly, in our cohort, 81% of women had severe preeclampsia and 55% preterm delivery consistent with more severe forms of the disease. These observations lead us to hypothesize that unlike normotensive pregnancies, where cardiac output increases early in pregnancy to support the placenta and fetus, in preeclampsia, the cardiovascular system is unable to adapt to the hemodynamic demands of pregnancy. The inability of the cardiovascular system to adapt to the early cardiovascular demands of pregnancy has been suggested to play a role in the pathophysiology of preeclampsia and warrants further investigation.^25^ Placenta-mediated abnormalities that increase maternal total vascular resistance are thought to be part of the pathophysiology that contributes to cardiovascular decompensation.^26,27^

Although we did not find a significant difference in J-amp × HR measures between the pregnancy and postpartum periods in the preeclampsia group, we did observe a trend toward an increase in J-amp × HR early in pregnancy, between the T1 and T2, followed by a drop that occurs in the T3.^28^ This trend, although not significant, is consistent with existing studies describing a hyperdynamic disease model in preeclampsia characterized by a drop in cardiac output with the development of preeclampsia, which may be represented by the drop in J-amp × HR observed in the T3.^29,30^ These observations lead us to hypothesize that this device may be able to identify the hemodynamic changes that occur before development of preeclampsia. It is important to note that antihypertensive medications could have contributed to the trend we noted in the T3 since medications commonly used to treat high blood pressure in preeclampsia, such as beta-blockers, can decrease cardiac output and contractility.

This pilot work has implications for future studies and clinical practice. In this study, we showed that the use of this noninvasive device is feasible in the outpatient clinic and inpatient settings similar to the time it takes to obtain vital signs. Future studies are needed to explore whether this device can identify hemodynamic changes that occur before development of preeclampsia, as suggested by our findings. Therefore, it could be used to identify women who should be started on preventive treatments such as aspirin. With advances in technology, the BCG signal can now be measured using a wearable device, which increases the clinical applications of this technology, as was seen in our study on HF patients.^11,12,31,32^

Our pilot study has strengths and limitations that are noteworthy. This pilot was the first study to assess cardiovascular hemodynamics noninvasively and longitudinally at numerous times points during pregnancy, prepartum, and postpartum in women with preeclampsia. BCG provides information on relative changes in cardiac output, and not absolute cardiac output, and therefore can be used to compare changes in BCG in participants; however, the ability to directly compare absolute BCG values from one subject to another is limited. Furthermore, because participants were enrolled during pregnancy, we do not have a baseline measure, limiting our ability to report the change from baseline for each participant. Although it is convenient to capture data in less than 2 minutes by standing on this modified weighing scale, the standing position limits our ability to compare with data obtained using validated tools in the recumbent position, and the measures may be affected by orthostasis.^33,34^

In addition, pedal edema may dampen the amplitude response of the signal, for example, a reduction in J-amp, which may indicate a relative reduction in stroke volume. However, as recording BCG with the modified scale has shown effectiveness in tracking hemodynamics and the clinical status in patients with HF,^11,12^ we have translated the same methods in tracking hemodynamics in pregnancy complicated with preeclampsia for this proof-of-concept study. Furthermore, in our pilot study, 25% of the women enrolled with suspected preeclampsia did not meet the criteria on chart review and therefore were not included in the final analysis. Because this was a pilot proof-of-concept study, we were limited by the number of women and particularly T1 data. Therefore, we did not have the power to evaluate differences in hemodynamic changes based on the severity of disease within preeclampsia (*i.e.*, preeclampsia vs. severe preeclampsia vs. chronic hypertension with superimposed preeclampsia). In addition, given the small sample size, we were unable to control for differences in baseline characteristics that would have affected hemodynamics, such as BMI, which has been associated with higher cardiac output^35^ and blood pressure medications used in most women with preeclampsia.

## Conclusions

We noninvasively measured cardiac hemodynamics throughout pregnancy in the clinical setting similar to the time it takes to obtain vital signs. We observe cardiac hemodynamic changes consistent with prior studies using well-validated invasive and noninvasive imaging modalities. In pregnancies complicated by preeclampsia, we observed that the maximal force of contraction was lower, suggesting lower cardiac output in pregnancies complicated by preeclampsia. We also observed a trend in J-amp × HRs in women with preeclampsia consistent with the hyperdynamic disease model. In future work, we will also explore the use of BCG to identify hemodynamic changes that precede development of preeclampsia.

## Supplementary Material

Supplemental data

Supplemental data

## Abbreviations Used

BCG: Ballistocardiogram
BMI: body mass index
ECG: electrocardiogram
E-PP: early postpartum
HF: heart failure
HR: heart rate
I-PP: immediate postpartum
IVF: *in vitro* fertilization
J-amp: J-wave amplitude
L-PP: late postpartum
T1: first trimester
T2: second trimester
T3: third trimester

## References

[1] Cirillo PM, Cohn BA. Pregnancy complications and cardiovascular disease death: 50-year follow-up of the Child Health and Development Studies pregnancy cohort. Circulation 2015;132:1234–1242 26391409 10.1161/CIRCULATIONAHA.113.003901 PMC6938224

[2] Skjaerven R, Wilcox AJ, Klungsoyr K, et al. Cardiovascular mortality after pre-eclampsia in one child mothers: Prospective, population based cohort study. BMJ 2012;345:e7677 23186909 10.1136/bmj.e7677 PMC3508198

[3] Wu P, Haththotuwa R, Kwok CS, et al. Preeclampsia and future cardiovascular health: A systematic review and meta-analysis. Circ Cardiovasc Qual Outcomes 2017;10:e003497 28228456 10.1161/CIRCOUTCOMES.116.003497

[4] Hypertension in Pregnancy. Report of the American College of Obstetricians and Gynecologists' Task Force on Hypertension in Pregnancy. Obstet Gynecol 2013;122:1122–1131 24150027 10.1097/01.AOG.0000437382.03963.88

[5] Inan OT, Migeotte PF, Park KS, et al. Ballistocardiography and seismocardiography: A review of recent advances. IEEE J Biomed Health Inform 2015;19:1414–1427 25312966 10.1109/JBHI.2014.2361732

[6] Kim CS, Ober SL, McMurtry MS, et al. Ballistocardiogram: Mechanism and potential for Unobtrusive Cardiovascular Health Monitoring. Sci Rep 2016;6:31297 27503664 10.1038/srep31297 PMC4977514

[7] Inan OT, Etemadi M, Wiard RM, et al. Robust ballistocardiogram acquisition for home monitoring. Physiol Meas 2009;30:169–185 19147897 10.1088/0967-3334/30/2/005

[8] Brüser C, Winter S, Leonhardt S. Robust inter-beat interval estimation in cardiac vibration signals. Physiol Meas 2013;34:123 23343518 10.1088/0967-3334/34/2/123

[9] Conn NJ, Schwarz KQ, Borkholder DA. In-home cardiovascular monitoring system for heart failure: Comparative study. JMIR Mhealth Uhealth 2019;7:e12419 30664492 10.2196/12419 PMC6356186

[10] Inan OT, Etemadi M, Paloma A, et al. Non-invasive cardiac output trending during exercise recovery on a bathroom-scale-based ballistocardiograph. Physiol Meas 2009;30:261–274 19202234 10.1088/0967-3334/30/3/003

[11] Aydemir VB, Nagesh S, Shandhi MMH, et al. Classification of decompensated heart failure from clinical and home ballistocardiography. IEEE Trans Biomed Eng 2019;67:1303–1313 31425011 10.1109/TBME.2019.2935619 PMC7271768

[12] Tracking clinical status for heart failure patients using ballistocardiography and electrocardiography signal features. 2014 36th Annual International Conference of the IEEE Engineering in Medicine and Biology Society, IEEE, 2014 10.1109/EMBC.2014.6944794 PMC4600348 25571162

[13] Vonck S, Staelens AS, Bollen I, et al. Why non-invasive maternal hemodynamics assessment is clinically relevant in early pregnancy: A literature review. BMC Pregnancy Childbirth 2016;16:302 27729024 10.1186/s12884-016-1091-9 PMC5059982

[14] Inan OT, Etemadi M, Wiard RM, et al. Novel methods for estimating the ballistocardiogram signal using a simultaneously acquired electrocardiogram. Conference proceedings: Annual International Conference of the IEEE Engineering in Medicine and Biology Society IEEE Engineering in Medicine and Biology Society Annual Conference 2009, 2009:5344–5347 10.1109/IEMBS.2009.5333709 19964385

[15] Hunter S, Robson SC. Adaptation of the maternal heart in pregnancy. Br Heart J 1992;68:540–543 1467047 10.1136/hrt.68.12.540 PMC1025680

[16] Melchiorre K, Sharma R, Khalil A, et al. Maternal cardiovascular function in normal pregnancy: Evidence of maladaptation to chronic volume overload. Hypertension 2016;67:754–762 26962206 10.1161/HYPERTENSIONAHA.115.06667

[17] Sanghavi M, Rutherford JD. Cardiovascular physiology of pregnancy. Circulation 2014;130):1003–1008 25223771 10.1161/CIRCULATIONAHA.114.009029

[18] Duvekot JJ, Peeters LL. Maternal cardiovascular hemodynamic adaptation to pregnancy. Obstet Gynecol Surv 1994;49(12 Suppl):S1–S14 7877788 10.1097/00006254-199412011-00001

[19] Ouzounian JG, Elkayam U. Physiologic changes during normal pregnancy and delivery. Cardiol Clin 2012;30:317–329 22813360 10.1016/j.ccl.2012.05.004

[20] Sabban H, Zakhari A, Patenaude V, et al. Obstetrical and perinatal morbidity and mortality among in-vitro fertilization pregnancies: A population-based study. Arch Gynecol Obstet 2017;296:107–113 28547098 10.1007/s00404-017-4379-8

[21] Spradley FT. Metabolic abnormalities and obesity's impact on the risk for developing preeclampsia. Am J Physiol Regul Integr Comp Physiol 2017;312:R5–r12 27903516 10.1152/ajpregu.00440.2016 PMC5283940

[22] Di Pasquo E, Ghi T, Dall'Asta A, et al. Maternal cardiac parameters can help in differentiating the clinical profile of preeclampsia and in predicting progression from mild to severe forms. Am J Obstet Gynecol 2019;221:633.e1–e33.e9. 31226294 10.1016/j.ajog.2019.06.029

[23] Visser W, Wallenburg HC. Central hemodynamic observations in untreated preeclamptic patients. Hypertension 1991;17(6 Pt 2):1072–1077 2045151 10.1161/01.hyp.17.6.1072

[24] Valensise H, Vasapollo B, Gagliardi G, et al. Early and late preeclampsia: Two different maternal hemodynamic states in the latent phase of the disease. Hypertension 2008;52:873–880 18824660 10.1161/HYPERTENSIONAHA.108.117358

[25] Kalafat E, Thilaganathan B. Cardiovascular origins of preeclampsia. Curr Opin Obstet Gynecol 2017;29:383–389 28961633 10.1097/GCO.0000000000000419

[26] Vasapollo B, Novelli GP, Valensise H. Total vascular resistance and left ventricular morphology as screening tools for complications in pregnancy. Hypertension 2008;51:1020–1026 18259001 10.1161/HYPERTENSIONAHA.107.105858

[27] Melchiorre K, Sutherland G, Sharma R, et al. Mid-gestational maternal cardiovascular profile in preterm and term pre-eclampsia: A prospective study. BJOG 2013;120:496–504 23190437 10.1111/1471-0528.12068

[28] Davis EF, Lewandowski AJ, Leeson P. Cardiac dysfunction and preeclampsia: Can imaging give clues to mechanism? Circ Cardiovasc Imaging 2012;5:691–692 23169981 10.1161/CIRCIMAGING.112.979831

[29] Bosio PM, McKenna PJ, Conroy R, et al. Maternal central hemodynamics in hypertensive disorders of pregnancy. Obstet Gynecol 1999;94:978–984. [published Online First: 1999/11/27] 10576186 10.1016/s0029-7844(99)00430-5

[30] Easterling TR, Benedetti TJ, Carlson KC, et al. The effect of maternal hemodynamics on fetal growth in hypertensive pregnancies. Am J Obstet Gynecol 1991;165(4 Pt 1):902–906 1951551 10.1016/0002-9378(91)90436-u

[31] Inan OT, Baran Pouyan M, Javaid AQ, et al. Novel wearable seismocardiography and machine learning algorithms can assess clinical status of heart failure patients. Circ Heart Fail 2018;11:e004313 29330154 10.1161/CIRCHEARTFAILURE.117.004313 PMC5769154

[32] Shandhi MMH, Hersek S, Fan J, et al. Wearable patch based estimation of oxygen uptake and assessment of clinical status during cardiopulmonary exercise testing in patients with heart failure. J Card Fail 2020;26:948–958 32473379 10.1016/j.cardfail.2020.05.014 PMC7704799

[33] Clark SL, Cotton DB, Pivarnik JM, et al. Position change and central hemodynamic profile during normal third-trimester pregnancy and post partum. Am J Obstet Gynecol 1991;164:883–887 2003555 10.1016/s0002-9378(11)90534-1

[34] Easterling TR, Schmucker BC, Benedetti TJ. The hemodynamic effects of orthostatic stress during pregnancy. Obstet Gynecol 1988;72:550–552 3419734

[35] Stelfox HT, Ahmed SB, Ribeiro RA, et al. Hemodynamic monitoring in obese patients: The impact of body mass index on cardiac output and stroke volume. Crit Care Med 2006;34:1243–1246 16484893 10.1097/01.CCM.0000208358.27005.F4

