# Supplementary material for: Use of Ballistocardiography to Monitor Cardiovascular Hemodynamics in Preeclampsia
Source: Womens Health Rep (New Rochelle). 2021 Apr 20;2(1):97–105. doi: 10.1089/whr.2020.0127 (PMC8080913; doi:10.1089/whr.2020.0127)
Supplement: Supplemental data [file Supp_Table1.docx]

**Supplemental Table 1. Blood Pressure by trimester in pregnancy and postpartum period in preeclampsia and normotensive control pregnancies**

| **Time in pregnancy and postpartum** | **Normotensive Controls (N=23)** | **Preeclampsia (N=30)** | **p-value** |  |
| --- | --- | --- | --- | --- |
|  |  |  |  |  |
| **First Trimester (T1)** |  |  |  |  |
| **SBP (mmHg)** | **111±13** | **125±5** | **0.02** |  |
| **DBP (mmHg)** | **65±7** | **72±9** | **0.32** |  |
| **Second Trimester (T2)** |  |  |  |  |
| **SBP (mmHg)** | **109±9** | **131±17** | **0.01** |  |
| **DBP (mmHg)** | **63±7** | **78±10** | **0.01** |  |
| **Third Trimester (T3)** |  |  |  |  |
| **SBP (mmHg)** | **114±9** | **133±10** | **<0.001** |  |
| **DBP (mmHg)** | **66±6** | **77±9** | **<0.001** |  |
| **Immediate Postpartum (I-PP)** |  |  |  |  |
| **SBP (mmHg)** | **111±7** | **131±11** | **<0.001** |  |
| **DBP (mmHg)** | **67±6** | **79±9** | **<0.001** |  |
| **Early Postpartum (E-PP)** |  |  |  |  |
| **SBP (mmHg)** | **112±11** | **123±11** | **0.003** |  |
| **DBP (mmHg)** | **69±6** | **76±12** | **0.02** |  |
| **Late Postpartum (L-PP)** |  |  |  |  |
| **SBP (mmHg)** | **105±5** | **118±16** | **0.05** |  |
| **DBP (mmHg)** | **66±6** | **79±13** | **0.04** |  |

***Values shown are mean ± standard deviation. SBP, systolic blood pressure, DBP, diastolic blood pressure.**
