# Supplementary material for: Use of Ballistocardiography to Monitor Cardiovascular Hemodynamics in Preeclampsia
Source: Womens Health Rep (New Rochelle). 2021 Apr 20;2(1):97–105. doi: 10.1089/whr.2020.0127 (PMC8080913; doi:10.1089/whr.2020.0127)
Supplement: Supplemental data [file Supp_Table2.docx]

**Supplemental Table 2. Number of women and recordings per trimester and postpartum period**

| **Recording Time** | **Normotensive Controls** | **Preeclampsia** |
| --- | --- | --- |
| **First Trimester (T1)** | **11 (13)** | **3 (4)** |
| **Second Trimester (T2)** | **17 (32)** | **7 (16)** |
| **Third Trimester (T3)** | **20 (83)** | **28 (69)** |
| **Immediate Postpartum (I-PP)** | **17 (22)** | **21 (26)** |
| **Early Postpartum (E-PP)** | **19 (19)** | **22 (31)** |
| **Late Postpartum (L-PP)** | **5 (10)** | **7 (10)** |

***Values are shown as number of subjects (number of recordings).**
